# Assessing the quality of medical death certification: a case study of concordance between national statistics and results from a medical record review in a regional hospital in the Philippines

**DOI:** 10.1186/s12963-018-0178-0

**Published:** 2018-12-29

**Authors:** Marilla Lucero, Ian Douglas Riley, Riley H. Hazard, Diozele Sanvictores, Veronica Tallo, Dorothy Gay Marmita Dumaluan, Juanita M. Ugpo, Alan D. Lopez

**Affiliations:** 1 Research Institute for Tropical Medicine, Muntinlupa City, Philippines; 2 School of Population and Global Health, University of Melbourne, Parkville, VIC Australia; 3 Philippine Academy of Family Physicians, Manila, Philippines; 4 Ramiro Community Hospital, Tagbilaran City, Philippines; 5 Holy Name University Medical Center, Bohol, Philippines

**Keywords:** Philippines, Medical record review, Cause of death, Medical certificates of cause of death, Vital registration

## Abstract

**Background:**

Medical certificates of cause of death (MCCOD) issued by hospital physicians are a key input to vital registration systems. Deaths certified by hospital physicians have been implicitly considered to be of high quality, but recent evidence suggests otherwise. We conducted a medical record review (MRR) of hospital MCCOD in the Philippines and compared the cause of death concordance with certificates coded by the Philippines Statistics Authority (PSA).

**Methods:**

MCCOD for adult deaths in Bohol Regional Hospital (BRH) in 2007–2008 and 2011 were collected and reviewed by a team of study physicians. Corresponding MCCOD coded by the PSA were linked by a hospital identifier. The study physicians wrote a new MCCOD using the patient medical record, noted the quality of the medical record to produce a cause of death, and indicated whether it was necessary to change the underlying cause of death (UCOD). Chance-corrected concordance, cause-specific mortality fraction (CSMF) accuracy, and chance-corrected CSMF were used to examine the concordance between the MRR and PSA.

**Results:**

A total of 1052 adult deaths were linked between the MRR and PSA. Median chance-corrected concordance was 0.73, CSMF accuracy was 0.85, and chance-corrected CSMF accuracy was 0.58. 74.8% of medical records were deemed to be of high enough quality to assign a cause of death, yet study physicians indicated that it was necessary to change the UCOD in 41% of deaths, 82% of which required addition of a new UCOD.

**Conclusions:**

Medical records were generally of sufficient quality to assign a cause of death and concordance between the PSA and MRR was reasonably high, suggesting that routine mortality statistics data are reasonably accurate for describing population level causes of death in Bohol. While overall agreement between the PSA and MRR in major cause groups was sufficient for public health purposes, improvements in death certification practices are recommended to help physicians differentiate between treatable (immediate) COD and COD that are important for public health surveillance.

**Electronic supplementary material:**

The online version of this article (10.1186/s12963-018-0178-0) contains supplementary material, which is available to authorized users.

## Background

Detailed and complete vital registration (VR) systems are important for effectively informing public health planning [1, 2]. Many countries rely on hospital deaths to update VR systems [1]. It is generally believed that hospital physicians have a comprehensive diagnostic understanding of their patients, and this will be reflected in high quality hospital cause of death (COD) statistics [3, 4].

A recent systematic review could identify only 29 studies that reported the accuracy of hospital data on COD published between 1980 and 2013 [5]. Importantly, most studies reported substantial misdiagnosis of all-cause mortality. The reviewers concluded that the assumption of high levels of accuracy in hospital COD data was unfounded. Other studies have identified poor death certification practices as a major issue [6]. For example, even after an extensive training period, Bangladeshi physicians failed to adhere to international standards in completing the medical certificate such as using ill-defined causes of death [7]. Poor adherence to medical certification practices can lead to COD statistics of uncertain value for VR systems and public health interventions.

Because of the implications for national health policy and planning, it is in the national interest for a country to periodically review the accuracy of COD data through a medical record review (MRR). Unfortunately, MRRs are not a routine part of hospital practice and have required external inputs from national departments of health to find out where education and training would best be directed [6].

A Medical Certificate of Cause of Death (MCCOD) is divided into two parts. Part 1 contains the sequence of causes that led to death; Part 2 contains conditions that contributed to the death but were not part of the sequence. The sequence in Part 1 establishes the underlying cause of death (UCOD) which is of principal interest to public health. Trained mortality coders not only assign codes from the International Classification of Diseases (10th edition) (ICD-10) to the conditions in the MCCOD, but also correct the sequence of causes according to the rules of the ICD-10 coding manual [8, 9].

In the Philippines, 35–45% of deaths occur in hospitals, depending on the region [10]. The MCCOD is written by a hospital physician familiar with the events leading to the patient’s death. This first-hand knowledge is supported by the contents of the medical record. Hospitals forward the completed MCCOD to the Office of the Civil Registrar which is part of the Philippines Statistics Authority (PSA), formerly the National Statistics Office. The certificates are then mortality coded and analyzed by the PSA. The results are reported to, as well as are published by, the Philippines Department of Health. The Department is an end-user of these statistics which it reports to the World Health Organization.

The Philippines has a mature and functioning VR system, but there has been little research about the accuracy of the UCOD assigned by the PSA [10–12]. To our knowledge, no study has examined the concordance between hospital deaths and those coded by the PSA. In this paper, we compared the UCOD reported by the PSA to that assigned by MRR at a regional hospital in the Philippines in order to assess the diagnostic accuracy of the PSA data.

## Methods

### Study overview

The study is based on adult, child, and neonate deaths in Bohol Regional Hospital (BRH), a Philippine public hospital, in 2007–2008 and 2011. The 2007 and 2008 data were collected as part of the Population Health Metrics Research Consortium (PHMRC) gold standard verbal autopsy validation study [13]. The 2011 data were collected as part of a study funded by the National Health and Medical Research Council (NHMRC) to determine statistical relationships between hospital and population mortality patterns by cause of death (grant no. 631494). Data were categorized and analyzed using methods developed for the PHMRC study.

### PHMRC gold standard cause of death categories

COD categories in this study were based on the cause lists developed for the validation of verbal autopsies in the PHMRC study, which had been based on WHO estimates of the leading causes of death in the developing world [13, 14]. The categories are mutually exclusive, collectively exhaustive, and can be coded to ICD-10 categories. Separate cause lists were developed for adults (≥12 years), children (28 days – 11 years) and neonates (< 28 days). The adult cause list used for the initial analysis is based on the 34 COD categories reported in an earlier publication [15].

### PHMRC gold standard criteria for assigning cause of death

GS criteria for assigning the UCOD were also based on those developed for the PHMRC Study [13]. The PHMRC GS criteria classified deaths into three levels based on the degree to which the information from the medical record provided sufficient certainty to determine whether the death could be used as part of the VA validation study: GS1, GS2A, and GS2B (high quality). GS1 diagnoses provide the highest level of diagnostic certainty possible for that condition, consisting of either an appropriate laboratory test or x-ray with positive findings, as well as medically observed and documented illness signs. GS2A diagnoses are of a high level of diagnostic certainty, consisting of medically observed and documented illness signs. GS2B was developed for chronic conditions where the original records were not available where records of treatment schedules were available from a reputable hospital. The NHMRC Study, which was concerned with all deaths in hospital, introduced two more levels (low quality): GS3 diagnoses relate to medical or health worker diagnoses not supported by the appropriate level of investigation, but which meet established clinical criteria, and GS4 diagnoses are unsupported by adequate clinical evidence.

Additional file 1 describes the gold standards for hospital diagnosis of the COD categories used in this study for adults. Additional file 2 shows investigations available to hospital patients in the BRH. Certain investigations (i.e., CT scan) were only available from private service providers outside the hospital.

### Medical record review

**Fig. 1:**
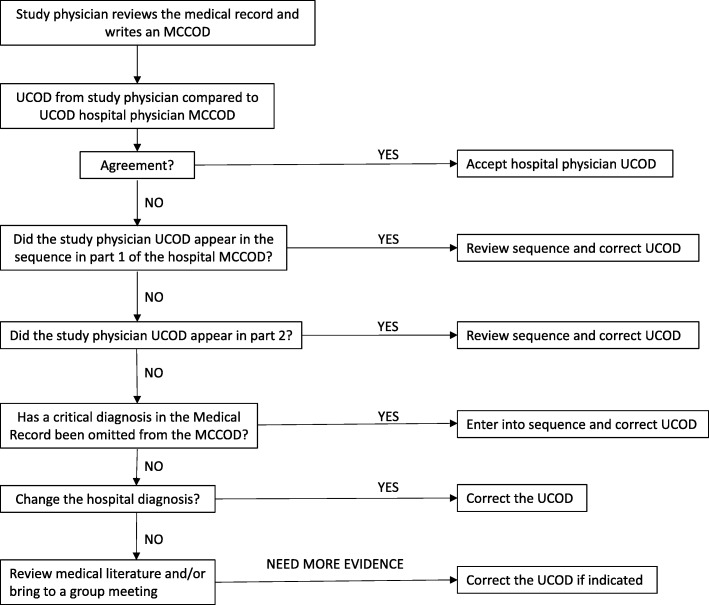
Medical record review process

A medical record review (MRR) depends solely on the content of the medical record to provide as accurate a diagnosis as possible. Trained study physicians at BRH reviewed hospital medical records which included the MCCOD written by a hospital physician. The steps taken in the medical review process are shown in Fig. 1. First, if there was agreement about the UCOD between study and hospital physicians, no further action was necessary. Second, if there was no agreement but the review UCOD appeared elsewhere in the hospital MCCOD, the review MCCOD was accepted. Third, if there was no agreement and the review UCOD did not appear in the hospital MCCOD, the review physician searched the medical record to see if the key diagnosis had been omitted from the MCCOD; in this case the key diagnosis replaced the hospital UCOD. Fourth, if the review physician rejected the hospital diagnoses completely, the rejection of the diagnosis and the introduction of a new diagnosis were based on disease definitions and descriptions in The Merck Manual of Diagnosis and Therapy [15]. Problem cases were discussed, and resolved, at group meetings.

The MCCOD written by the hospital physicians were forwarded to the Tagbilaran City Civil Registrar and transmitted to the Office of the Civil Registrar General where they were mortality coded according to the rules of the ICD-10 [9]. The coded medical certificates were analyzed by the PSA.

**Fig. 2:**
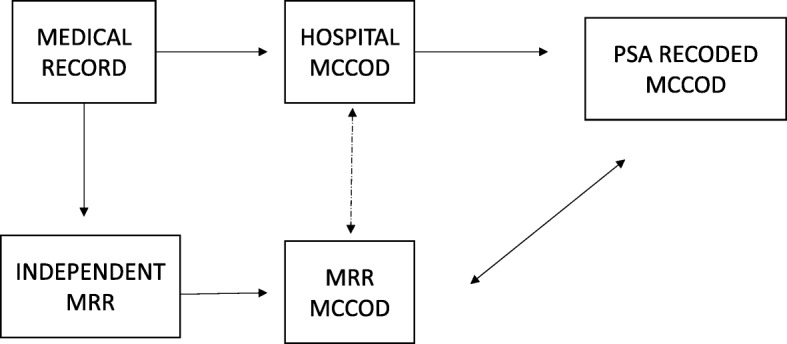
Process of comparing the medical certificates of cause of death (MCCOD) from the medical record review (MRR) and Philippine Statistics Authority (PSA)

Both hospital physician and MRR physician diagnoses were subsequently recoded to the PHMRC gold standard COD categories described above. MRR UCOD were then linked to PSA MCCOD by a hospital registration number. The UCOD assigned by the study physician and the PSA were then compared for each individual death in the sample (Fig. 2).

### Training and supervision of study physicians

Study physicians were trained on the job by two of the authors (ML and IR). Study physicians were supervised at regular intervals by these authors who reviewed their results and discussed problem cases with them. The same two physicians continued as reviewers through the course of the study.

### Analysis: change of UCOD

As part of the review process, the reviewers recorded:

1. whether it had been necessary to change the UCOD and if it had been changed,
2. whether the change to the UCOD was due to a change in diagnoses appearing on the MCCOD, and
3. whether change to the UCOD was due to changes made to the sequence of causes leading to the UCOD.

The reviewers were asked to record major changes only. In practice, change was only recorded at the ICD-10 three-digit level. For example, I21.0 Acute transmural myocardial infarction of anterior wall and I21.9 Acute myocardial infarction, unspecified would both be coded to I21Acute myocardial infarction. These rules were not absolute and were not applied if the investigative capacity of the hospital failed to meet requirements at this level of the ICD. For example, I61 Intracerebral hemorrhage and I63 Cerebral infarction were both coded as I64 Stroke.

### Analysis: misclassification matrix

We created a misclassification matrix to compare agreement between sources for individual CODs. The matrix was initially based on the PHMRC GS list of 34 adult causes but reduced to 22 causes because of a small number of deaths (< 7) in certain causes.

### Analysis: performance metrics

To assess agreement between the UCOD assigned by MRR and the PSA, we varied the cause-specific mortality fraction (CSMF) compositions of the MRR to avoid estimates that were biased by only a single CSMF composition [16]. We created 500 random splits of the MRR dataset, and for each split we sampled from a Dirichlet function to produce a new cause distribution that was independent of any other cause distribution and based only on the causes of death in the MRR dataset. We then randomly sampled from the MRR and PSA dataset, stratified by MRR causes, so that the strata matched the cause distribution from the random cause distribution of the Dirichlet function.

For each split, we calculated chance-corrected concordance (CCC) for each cause. CCC is a measure of agreement between the MRR and PSA cause assignment adjusted for chance [16]. We also calculated CSMF accuracy and chance corrected cause specific mortality fraction (CCCSMF) accuracy for each split [17]. CSMF accuracy is a summary measure of performance between the MRR and PSA cause assignment, and CCCSMF adjusts for chance. This procedure was calculated for all deaths, high quality diagnoses (GS1, GS2A, and GS2B), and low quality diagnoses (GS3 and GS4).

## Results

### Study sample and outcome of the physician review

A total of 1749 and 1241 deaths were collected from MRR and PSA, respectively, with most deaths occurring in adults. Table 1 shows the numbers of deaths subject to MRR, their distribution by GS and the number of corresponding records retrieved from the PSA. One thousand fifty-two adult deaths, 92 child deaths, and 97 neonate deaths were available for analysis. Approximately three-fourths of deaths were classified as having high quality diagnoses (GS1, 2A, and 2B) and the remainder as low quality diagnoses (GS3 and 4) across all age groups. Because of low numbers of child and neonate deaths, the subsequent analyses of UCOD focused on adult deaths.

**Table 1:** All deaths in the Bohol Regional Hospital by age group, GS level, and source of UCOD

GS Level	Adult			Child			Neonate			Total		
	MRR	PSA	%	MRR	PSA	%	MRR	PSA	%	MRR	PSA	%
GS 1	551	498	47.3	75	46	50	197	46	47.4	823	590	47.5
GS 2A	299	270	25.7	31	19	20.7	55	19	19.6	385	308	24.8
GS 2B	18	17	0.2	4	2	2.2	0	0	0	22	19	1.5
GS 3	213	183	17.4	34	19	20.7	64	16	16.5	311	218	17.6
GS 4	77	68	6.5	8	4	4.3	22	4	4.1	107	77	6.2
Other	21	16	1.5	5	2	2.2	75	12	12.4	101	29	2.3
Total	1179	1052	100	157	92	100	413	97	100	1749	1241	100

Abbreviations: *GS* Gold standard, *MRR* Medical record review, *PSA* Philippines statistics authority

### Change of UCOD

The study physicians reported that they changed the UCOD at the three-digit level in 41.2% (432/1049) of the MCCOD. The change was due to a change in the sequence of causes in 7.2% of all MCCOD, and to the introduction of a new diagnosis in 33.7% of the MCCOD. Thus, 82% of changes in the UCOD were due to a failure to include a correct diagnosis. Reported percentage changes are based on three-digit ICD-10 categories as described above; missing values in 6/1052 deaths.

### Misclassification matrix

Additional file 3 shows a misclassification matrix for all adult deaths comparing UCOD derived from the MRR with PSA. Specific causes with less than seven deaths identified by the study physicians were incorporated into residual categories for purposes of analysis. The table shows concordance for 16 specific causes and five residual categories (other non-communicable diseases [other NCDs], other infections, other cardiovascular [other CVS], other cancers, and other injuries). Overall, simple concordance for adult deaths between the two sources was 69.2%. Agreement between diagnoses for high quality adult diagnoses was 70.6% (Additional file 4) and 64.5% for low quality diagnoses (Additional file 5).

Overall agreement for pneumonia was low. Deaths identified as being caused by pneumonia by the MRR were allocated, in particular, to pulmonary tuberculosis, stroke, diabetes, and residual categories. Pneumonia deaths identified by the PSA were allocated to pulmonary tuberculosis, renal failure, stroke, and other NCDs. Stroke deaths identified by the MRR were allocated to diabetes, other cardiovascular diseases, other NCDs, and pneumonia.

**Fig. 3:**
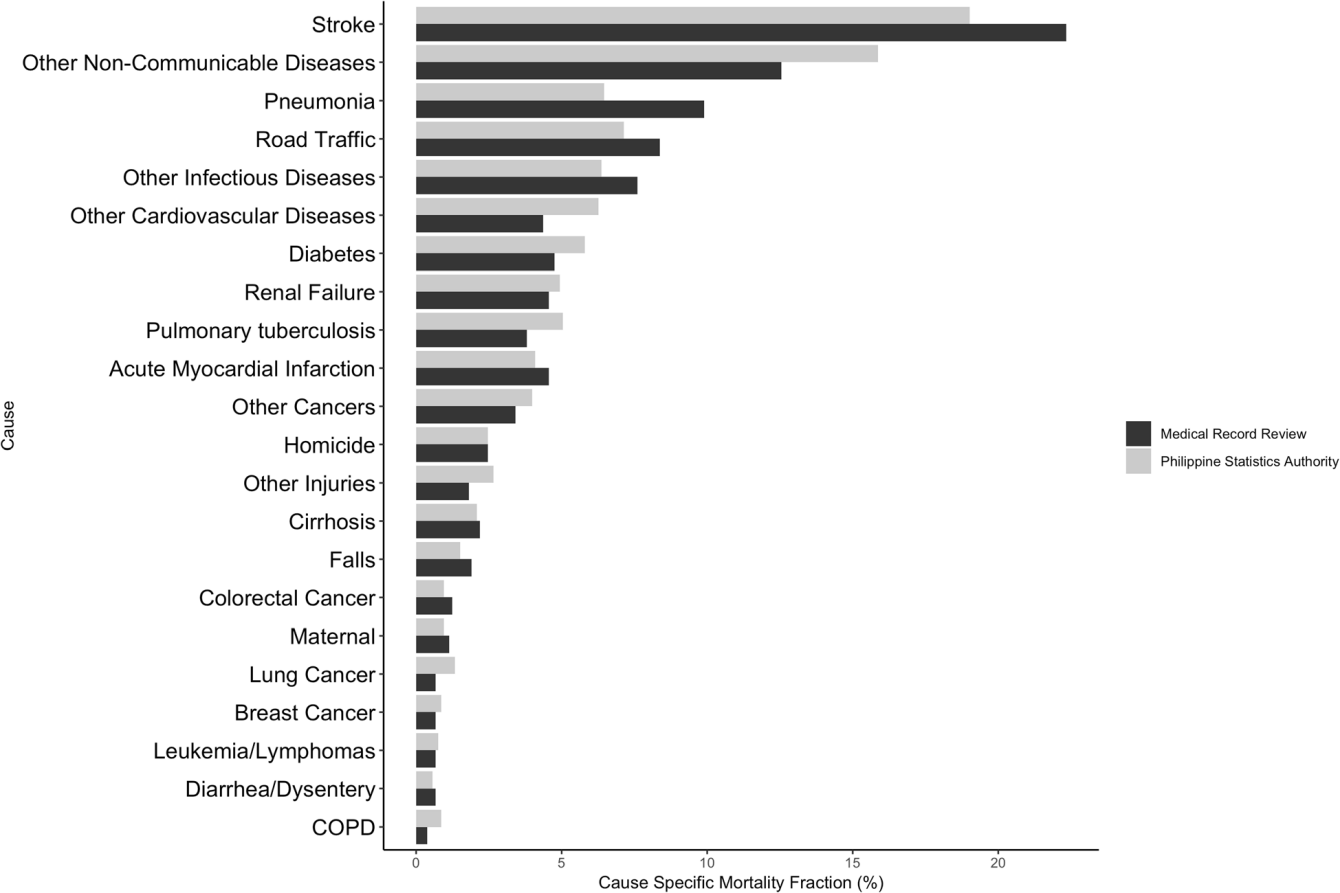
Cause-specific mortality fractions for deaths assigned by medical record review (MRR) and by the Philippines Statistics Authority (PSA)

Overall, the CSMF distributions show broad agreement between the two sources (Fig. 3). Noteworthy differences include a higher percentage of deaths from strokes, pneumonia, road traffic injuries, maternal causes, and other infections in the results from the MRR; and higher percentage of deaths from pulmonary tuberculosis, lung cancer, other non-communicable diseases, other cardiovascular diseases, other cancers, and other injuries in the results from the PSA.

### Performance metrics

The performance metrics have been designed to assess agreement between the sources by removing bias due to variation in the distribution of causes. Median CCC, CSMF accuracy, and CCCSMF were reasonably high for all death record quality types (Table 2). The median CSMF accuracy was 0.85 and CCCSMF accuracy was 0.58 for all deaths, indicating that the CSMF for PSA showed relatively close agreement with that of the MRR in this sample. Low and high quality diagnoses did not show any noticeable difference in CSMF performance from all deaths.

**Table 2:** Agreement between medical record review (MRR) and Philippine Statistics Authority (PSA) cause of death

Median performance	Gold standard level					
	All		High quality		Low quality	
	Median	95% UI	Median	95% UI	Median	95% UI
CCC	0.73	(0.73,0.74)	0.74	(0.73,0.74)	0.73	(0.73,0.74)
CSMF accuracy	0.85	(0.84,0.85)	0.85	(0.84,0.85)	0.84	(0.84,0.85)
CCCSMF accuracy	0.58	(0.57,0.59)	0.58	(0.57,0.59)	0.57	(0.56,0.58)

Abbreviations: *UI* Uncertainty interval, *CCC* Chance-corrected concordance, *CSMF* Cause-specific mortality fraction, *CCCSMF* Chance-corrected cause specific mortality fraction

## Discussion

After reviewing medical records at the BRH over 3 years, study physicians found it necessary to correct the UCOD at the three-digit level in 41.2% of the MCCOD; the change was due to a change in the sequence of causes in 7.2% of all MCCOD and to the introduction of a new diagnosis in 33.7% of these. Overall chance-corrected cause agreement between the MRR and PSA in the misclassification matrix (22 causes) was reasonably high at both the individual (0.73) and population level (0.58). The PSA reports the 10 leading causes of death in the country by sex [18]. The report covers about 70% of all causes and includes categories such as Neoplasms and Other heart conditions. Findings from this study in Bohol indicate that a similar report covering the leading 22 causes and including all hospital deaths would be reasonably accurate.

The individual and population level concordance between the MRR and the PSA was higher than would have been expected given the percentage of MCCOD that required revision of the UCOD. However, the level of concordance is correlated with the number of COD categories under consideration: the higher the number of categories, the lower the concordance. In reporting revisions to UCOD, the study physicians were dealing with a much greater number of causes than the final 22 aggregated categories. The fact that the concordance between the MRR and PSA was high despite a large proportion of the MCCOD requiring insertion of a new cause suggests that many of the causes inserted by the study physicians were in the same cause categories as the causes indicated by the PSA. Also, there was no significant difference between the concordance of high quality versus low quality diagnoses, which was likely due to low quality medical records providing so little information that the study physicians were required to come to the same UCOD conclusion as the hospital physician.

Overall, the quality of the Philippine medical records was comparatively high, with 75% of records classified as GS1, GS2A, or GS2B. That is, these records provided evidence to justify the study physician diagnosis with a reasonable degree of certainty. However, 41.2% of adult deaths required a change in the UCOD on the MCCOD and 82% of these deaths required introduction of a new COD to the certificate. It follows that either 1) the study physicians did not accept a key diagnosis and altered it on the basis of the clinical record or 2) a diagnosis was present in the medical record but had not been not entered into the MCCOD. The second explanation implies a fundamental failure in entering all diagnoses on the clinical record to the MCCOD. (In the remaining 18% of cases, either the UCOD had not been entered into Part 1 of the certificate or else the sequencing was incorrect.)

There are several possible explanations for these corrections. First, record maintenance and medical certification of deaths may be assigned to the most junior member of the clinical team because senior clinicians view medical death certification as tedious and boring. Second, it was observed that many clinical investigations were not attached to the medical record following the death of the patient and needed to be tracked back to the laboratory or the radiology department. The hospital physician would not necessarily have seen these results when completing the MCCOD. Third, the diagnoses that should have been included in the MCCOD and hence for VR were ignored in favor of diagnoses that were essential for clinical decision-making.

Several studies have examined concordance between the MCCOD and MRR, but this study is best compared with a study in Mexico by Hernandez et al. that used similar GS criteria, COD categories, and robust metrics [5, 19]. Hernandez et al. examined 1284 adult deaths in Mexico and compared the UCOD obtained from medical certificates against gold standard diagnoses developed by the PHMRC and derived from MRR. Median CCC increased from 66.5 to 75.9% when considering the mention of any COD on the death certificate. The median CCC for the UCOD in the Philippines was higher than in Mexico but slightly lower than that for all COD on the medical certificates in Mexico. The results of the Mexico study suggest that hospital physicians failed to correctly indicate the UCOD on the medical certificate in 33.5% of cases, but only 30% of these failures could be explained by physicians inputting the incorrect sequence of causes leading to death. 70% of the disagreements would have required the introduction of a new cause to the certificate. The situation is thus similar to the Philippines, where 41.0% of medical certificates required a change in UCOD, 18% were due to an incorrect sequence, and 82% of these were consequent upon the introduction of a new COD.

This study was part of a larger NHMRC study which brought with it several strengths. First, the study physicians in this study had experience in local settings as well as using and training others in MRR. Second, the number of cause categories (22) was appropriate to calculate concordance metrics. Too few categories are too broad for analysis and too many categories are too narrow. We recommend selecting 20–30 leading causes and establishing a standard list of causes for reviews in different hospitals. Third, this study calculated robust metrics for assessing concordance that avoid the bias of using a single CSMF composition.

It could be argued that medical reviewers should be blinded to the hospital physicians’ MCCOD. In our view this would not be helpful. The aim of the review is to produce the most accurate MCCOD possible. The hospital physician, with personal experience of the patient, has access to signs and symptoms denied the reviewer. Our practice was to establish the likely COD, criticize clinical diagnosis as appropriate, and to compare reviewer diagnoses with the hospital MCCOD. Records are not always well kept, and it is not difficult to miss a key point. Given that they changed the UCOD in over 40% of deaths, the Bohol reviewers were not inhibited in making changes.

Nonetheless, the present study has its limitations. The hospital data were based on a gold standard validation study for verbal autopsies. A UCOD was assigned by study physicians to each death following a MRR and a review of the hospital physician MCCOD. This study physician UCOD was compared with the UCOD assigned by the PSA. Both the study physicians and the PSA corrected the sequence of causes in the hospital physician MCCOD, but the PSA lacked access to the medical record. Differences between the two could either have been due to different revisions of the hospital physician sequences or to the addition of new causes obtained from the medical record. Because only the UCOD was coded by the validation study and the PSA, we were not able to compare the effects of the changes to sequence directly. The finding of failure to include all possible causes on the MCCOD had not been anticipated and required more detailed analysis than we could provide.

On the other hand, the evidence that 82% of the changes made to the hospital physician UCOD was a consequence of the failure to include all possible causes suggests very strongly that this was a fundamental problem. Residual, or other, categories had been developed in the gold standard validation studies for defined causes of death not specified elsewhere. The present study included not only high quality but also low quality diagnoses, which resulted in undefined causes being included in residual categories. Low counts of children and neonate deaths made it impossible to calculate appropriate metrics to measure concordance between the MRR and PSA assignment.

## Conclusions

Filipino physicians had difficulty in transferring the UCOD from the medical record to the MCCOD, but the UCOD reported by the PSA was generally in agreement with that of study physicians. This suggests that the routine mortality data from PSA, at least for Bohol, might be used with some confidence to describe comparative cause of death patterns in the population. These results suggest that medical records used to complete the MCCOD and inform the VR system in the Philippines are of sufficient quality in their assigning of an UCOD and most MCCOD are sufficient for public health purposes (i.e., to report the leading 10–20 causes of death in the country). Junior physicians are commonly assigned the tasks of maintaining clinical records and of writing MCCOD. If unsupervised in these tasks, they are open to making errors, firstly, in their interpretation of the correct clinical diagnosis and, secondly, in their entry of diagnoses into the MCCOD. Review of MCCOD needs to become part of routine clinical audits by hospital teams which can be used both to strengthen clinical practice and to improve the quality of medical records and MCCOD. Future studies that conduct MRRs should continue to use rigorous GS criteria and compute robust metrics to avoid bias.

## Additional files

Additional file 1: Standard for hospital diagnosis of cause of death in adults. Cause list and gold standard criteria for each adult cause of death. (DOCX 26 kb)

Additional file 2: Investigations and procedures available to patients in the Bohol Regional Hospital. Table that outlines the resources available for laboratory investigation at Bohol Regional Hospital. (DOCX 17 kb)

Additional file 3: Misclassification matrix table for all adult deaths. Misclassification matrix for gold standard 1 and 2 deaths that compares the underlying cause of death assigned by study physicians with the underlying cause of death assigned by the Philippine Statistics Authority. (XLSX 19 kb)

Additional file 4: Misclassification matrix for adult gold high quality diagnoses. Misclassification table for gold standard 1 and 2 deaths that compares the underlying cause of death assigned by study physicians with the underlying cause of death assigned by the Philippine Statistics Authority. (XLSX 15 kb)

Additional file 5: Misclassification matrix for adult gold low quality diagnoses. Misclassification table for gold standard 3 and 4 deaths that compares the underlying cause of death assigned by study physicians with the underlying cause of death assigned by the Philippine Statistics Authority. (XLSX 15 kb)

## Abbreviations

BRH: Bohol Regional Hospital
CCC: Chance-corrected concordance
CCCSMF: Chance-corrected cause-specific mortality fraction
COD: Cause of death
CSMF: Cause-specific mortality fraction
GS: Gold standard
ICD: International classification of diseases
MCCOD: Medical certificate of cause of death
MRR: Medical record review
NCDS: Non-communicable diseases
NHMRC: National Health and Medical Research Council
PHMRC: Population Health Metrics Research Consortium
PSA: Philippines Statistics Authority
UCOD: Underlying cause of death
UI: Uncertainty interval
VR: Vital registration
