# Supplementary material for: Assessing the quality of medical death certification: a case study of concordance between national statistics and results from a medical record review in a regional hospital in the Philippines
Source: Popul Health Metr. 2018 Dec 29;16:23. doi: 10.1186/s12963-018-0178-0 (PMC6311069; doi:10.1186/s12963-018-0178-0)
Supplement: Supplementary file 1 — Standard for hospital diagnosis of cause of death in adults. Cause list and gold standard criteria for each adult cause of death. (DOCX 26 kb) [file 12963_2018_178_MOESM1_ESM.docx]

| **Additional File 1** | |  |
| --- | --- | --- |
| **STANDARDS FOR HOSPITAL DIAGNOSIS OF CAUSE OF DEATH IN ADULTS** | | |
|  |  |  |
|  |  |  |
| **GS1: Highest level of certainty** | | Diagnosis of a particular condition with the highest level of certainty possible for that condition, consisting of an appropriate laboratory test or x-ray with positive findings and/or medically observed and documented appropriate illness sign(s). |
| **GS2A: High level of certainty** | | Diagnosis of a particular condition with a high level of certainty, consisting of an appropriate level of investigation and/or of medically observed and documented appropriate illness or sign(s). |
| **GS2B: High level of certainty** | | Presumed initial diagnosis of a particular condition with high certainty; this category was developed especially for cancer and HIV patients on long-term treatment where initial data had been lost |
| **GS3: Reasonable level of certainty** | | Medical or health worker diagnoses not supported by the appropriate level of investigation but which meet established clinical criteria |
| **GS4: Unsupported diagnosis** | | Medical diagnosis unsupported by adequate clinical evidence |
|  |  |  |
|  |  |  |
| **INFECTIOUS DISEASES** | | |
| **GC13 VA CODE** | **Level of Diagnosis** | **Criteria for Diagnosis** |
| **B20** | **AIDS with or without TB** | |
|  | **GS4** | Unsupported clinical diagnosis |
|  | **GS3** | AIDS defining condition [or generalised, unexplained lymphadenapothy] |
|  | **GS2B** | Receiving ARV therapy based on laboratory investigation in the past |
|  | **GS2A** | Routine rapid HIV test (or ELISA) +ve AND (generalised, unexplained lymphadenapothy OR AIDS defining condition) |
|  | **GS1** | Routine rapid HIV test (or ELISA) AND Western Blot +ve AND (generalised, unexplained lymphadenapothy OR AIDS defining condition) |
| **A091** | **DIARRHOEA** | |
|  | **GS4** | Unsupported clinical diagnosis |
|  | **GS1** | Observed dehydration AND (reported liquid or loose stools 3+ times a day for at least 1 day OR observed liquid stools) |
| **A092** | **DYSENTERY** | |
|  | **GS4** | Unsupported clinical diagnosis |
|  | **GS3** | Bloody diarrhea |
|  | **GS2A** | Bloody diarrhea with fever AND gripping abdominal pain AND tenesmus and/or rectal prolapse |
|  | **GS1** | Bloody diarrhoea AND *Shigella* culture+ve OR *E.histolytica* trophozoites in stools |

| **J12** | **PNEUMONIA** | |
| --- | --- | --- |
|  | **GS4** | Unsupported clinical diagnosis |
|  | **GS3** | Cough, dyspnoea, and an acute febrile illness |
|  | **GS2** | Cough AND two of: |
|  |  | Respiratory rate >20/min, , |
|  |  | Abnormal auscultatory findings |
|  |  | Fever |
|  | **GS1** | Clinical as for GS2 AND chest xray consolidation |
| **A15** | **PULMONARY TUBERCULOSIS** | |
|  | **GS4** | Unsupported clinical diagnosis |
|  | **GS3** | Clinical history consistent with active pulmonary tuberculosis during terminal illness AND typical chest xray findings |
|  | **GS1** | 1) Clinical history consistent with active pulmonary tuberculosis during terminal illness AND 2) Sputum smear or culture positive |
|  | **Excludes** chronic lung disease as a result of tuberculosi**s** | |
| **ZZ21** | **OTHER SPECIFIED INFECTIONS** | |
|  | In assigning a level of diagnostic certainty, the same general principles apply | |
| **CANCERS** | | |
|  |  |  |
| **C50** | **BREAST** | |
|  | **GS4** | Unsupported clinical diagnosis |
|  | **GS3** | Breast mass with enlarged lymph nodes or other clinical evidence of metastases |
|  | **GS2B** | Patient under treatment for breast cancer from a recognised cancer hospital or unit |
|  | **GS2A** | Breast mass and 1)Mammography typical AND 2) Imaging evidence of metastases |
|  | **GS1** | Breast mass with histological or cytological confirmation |
| **D05** | **CERVICAL** | |
|  | **GS4** | Unsupported clinical diagnosis |
|  | **GS2B** | Patient under treatment for cervical cancer from a recognised cancer hospital or unit |
|  | **GS2A** | Visualisation |
|  | **GS1** | Visualisation AND (Pap smear OR Cervical biopsy +ve) |
| **G18** | **COLO-RECTAL** |  |
|  | **GS4** | Unsupported clinical diagnosis |
|  | **GS2B** | Patient under treatment for colo-rectal cancer from a recognised cancer hospital or unit |
|  | **GS2A** | Endoscopy OR Imaging OR Report of laparotomy |
|  | **GS1** | Confirmed histology |
| **C15** | **ESOPHOGEAL** | |
|  | **GS4** | Unsupported clinical diagnosis |
|  | **GS3** | Oesophageal obstruction leading to death |
|  | **GS2B** | Patient under treatment for oesophageal cancer from a recognised cancer hospital or unit |
|  | **GS2A** | Barium contrast OR Endoscopy |
|  | **GS1** | Histology |

| **D91** | **LEUKEMIA** | |
| --- | --- | --- |
|  | **GS4** | Unsupported clinical diagnosis |
|  | **GS2B** | Patient under treatment for leukaemia from a recognised cancer hospital or unit |
|  | **GS2A** | Full blood count with hematology report |
|  | **GS1** | Bone marrow aspiration |
| **C34** | **LUNG** | |
|  | **GS4** | Unsupported clinical diagnosis |
|  | **GS2B** | Patient under treatment for lung cancer from a recognised cancer hospital or unit |
|  | **GS2A** | Bronchoscopic confirmation OR chest xray with single mass |
|  | **GS1** | Histological confirmation |
| **G96** | **LYMPHOMA** | |
|  | **GS4** | Clinical diagnosis |
|  | **GS2B** | Patient under treatment for lymphoma from a recognised cancer hospital or unit |
|  | **GS1** | Histological confirmation |
| **H61** | **PROSTATE** | |
|  | **GS4** | Clinical diagnosis |
|  | **GS3** | Nodular prostate and elevated PSA |
|  | **GS2B** | Patient under treatment for prostate cancer from a recognised cancer hospital or unit |
|  | **GS2A** | Elevated PSA AND (Ultrasound OR Xray of metastases) |
|  | **GS1** | Histological confirmation |
| **A16** | **STOMACH** | |
|  | **GS4** | Clinical diagnosis |
|  | **GS3** | Palpable mass; signs of obstruction |
|  | **GS2B** | Patient under treatment for stomach cancer from a recognised cancer hospital or unit |
|  | **GS2A** | Endoscopy OR imaging |
|  | **GS1** | Histological confirmation |
| **ZZ22** | **CANCERS FROM SITES OTHER THAN THE ABOVE (Residual category)** | |
|  | In assigning a level of diagnostic certainty, the same general principles apply: | |
|  | **GS4** | Unsupported clinical diagnosis (includes primary site unknown) |
|  | **GS3** | Primary site identified but lacks visual or surgical identification |
|  | **GS2B** | Patient under treatment from a recognised cancer hospital or cancer unit for cancer from a specific site other than the above in cases where the basis for the initial diagnosis is no longer available |
|  | **GS2A** | Visualisation of the cancer without further confirmation, e.g. diagnosis at laparotomy without histological confirmation |
|  | **GS1** | Histological confirmation |
|  | **NON-COMMUNICABLE DISEASES** | |
|  |  |  |
| **J45** | **ASTHMA** | |
|  | **GS4** | Unsupported clinical diagnosis |
|  | **GS3** | Status asthmaticus |
|  | **GS2A** | Status asthmaticus in known asthmatic |
|  | **GS1** | Status asthmaticus AND Spirometry or serial peak flow: reversible airway obstruction > 20% |

| **J33** | **COPD: CHRONIC OBSTRUCTIVE PULMONARY DISEASE** | |
| --- | --- | --- |
|  | **GS4** | Unsupported clinical diagnosis |
|  | **GS3** | Productive cough and breathlessness for 3 or more months of the year for a minimum of two successive years; terminal illness as above |
|  | **GS2A** | Productive cough and breathlessness for 3 or more months of the year for a minimum of two successive years ; medical diagnosis and treatment of acute exacerbations; terminal illness as above |
|  |  | Terminal illness due to pneumonia, cor pulmonale, or respiratory failure AND Chest xray shows hyperinflation |
|  | **GS1** | History typical of COPD AND Spirometry has shown FEV1 < 70% with no response to bronchodilators OR |
|  | **DIABETES (Screening of a healthy person)** | |
|  | **GS4** | Unsupported clinical diagnosis |
|  | **GS3** | Glucosuria |
|  | **GS1** | 1) Fasting glucose ≥7.0 mmol/L (≥126 mg/dl) OR 2) Oral glucose tolerance test: 2 hour glucose ≥11.1 mmol/L (≥200 mg/dl) OR 3) HbA1c>6.5 mg/dl |
|  | **DIABETES: HOSPITALISED FOR A COMPLICATION OF DIABETES BUT NO DOCUMENTED HISTORY** | |
|  | **GS4** | Unsupported clinical diagnosis |
|  | **GS3** | Persistent glucosuria or elevated blood glucose not meeting GS1 criteria |
|  | **GS1** | Random glucose ≥11.1 mmol/L (≥200 mg/dl) on at least two occasions not influenced by recent meal or intravenous glucose |
|  | **DIABETES: HOSPITALISED FOR A COMPLICATION BUT WITH DOCUMENTED HISTORY** | |
|  | **GS4** | Unsupported clinical diagnosis |
|  | **GS3** | Persistent glucosuria or blood glucose not meeting GS1 criteria |
|  | **GS1** | Random glucose ≥11.1 mmol/L (≥200 mg/dl) on at least **one** occasion not influenced by recent meal or intravenous glucose |
| **K71** | **HEPATIC CIRRHOSIS** | |
|  | **GS4** | Unsupported clinical diagnosis |
|  | **GS3** | Signs and symptoms of liver failure; no hepatic masses AND Signs and symptoms of liver failure |
|  | **GS2A** | Abormal liver function tests AND abnormal ultrasound or other imaging OR |
|  |  | Haematemesis AND endoscopic confirmation of oesophageal varices |
|  | **GS1** | Liver biopsy |
| **G40** | **EPILEPSY** | |
|  | **GS4** | Unsupported clinical diagnosis |
|  | **GS3** | Seizures leading to death not meeting the GS1 criteria |
|  | **GS1** | Status epilepticus in the absence of any underlying cause AND past history of seizures |
| **I21** | **IHD: ACUTE MYOCARDIAL INFARCTION** | |
|  | **GS4** | Unsupported clinical diagnosis |
|  | **GS3** | Characteristic history |
|  | **GS2A** | Sudden death within six hours of characteristic chest pain and shock witnessed by a physician |
|  | **GS1** | History consistent with infarct OR documented history of CABG or PTCA or stenting AND |
|  |  | ECG changes OR Enzyme changes (troponin elevation or CK-MB elevation > 2 by upper limit of normal in context of myocardial ischaemia) |

| **N17** | **RENAL FAILURE DUE TO RENAL DISEASE** | |
| --- | --- | --- |
|  | **GS4** | Unsupported clinical diagnosis |
|  | **GS3** | Elevated blood urea and/or serum creatinine; Diabetes not excluded |
|  | **GS2** | Elevated blood urea and/or serum creatinine; Diabetes excluded. If hypertension is associated with renal failure, the hypertension must be shown to be causal |
|  | **GS1** | Elevated serum creatinine; Renal biopsy or renal ultrasound or other evidence of intrinsic renal involvement such as proteinuria or urinary red blood cell casts. |
| **I64** | **STROKE** | |
|  | **GS4** | Unsupported clinical diagnosis |
|  | **GS2A** | Rapidly developing signs of focal or global loss of function |
|  | **GS1** | 1) Rapidly developing signs of focal or global loss of function AND |
|  |  | 2) CT Scan OR MRI |
| **ZZ23** | **OTHER SPECIFIED CARDIOVASCULAR DISEASES** | |
| **ZZ24** | **OTHER SPECIFIED DIGESTIVE DISEASES** | |
| **ZZ25** | **OTHER SPECIFIED NON-COMMUNICABLE DISEASES** | |
| In assigning a level of diagnostic certainty, the same general principles apply | | |
|  | **MATERNAL** | |
| A maternal death is the death of a woman during pregnancy or within 6 weeks of either abortion or birth. It is a death from any cause related to or aggravated by the pregnancy or its management, but not from accidental or incidental causes. | | |
| **W15** | **ANAEMIA** | |
|  | **GS4** | Unsupported clinical diagnosis |
|  | **GS2** | Congestive heart failure AND extreme pallour |
|  | **GS1** | Congestive heart failure AND Hb ≤ 3 |
| **O67** | **HAEMORRHAGE** | |
|  | **GS4** | Unsupported clinical diagnosis |
|  | **GS2A** | As for GS1. Blood loss not measured. |
|  | **GS1** | **Antepartum Haemorrhage**: Shock following measured blood loss > 500ml due to placenta praevia or placental separation OR |
|  |  | **Primary Post Partum Haemorrhage** (within 24 hours of vaginal delivery): Shock with measured blood loss > 500ml |
|  |  | **Secondary Post Partum Haemorrhage** (> 24 hours after vaginal delivery): Shock with measured blood loss > 500ml |
|  |  | **Spontaneous or medically induced abortion:** Shock with measured blood loss > 500ml |
| **H11** | **HYPERTENSIVE DISORDER (ECLAMPSIA)** | |
|  | **GS4** | Unsupported clinical diagnosis |
|  | **GS3** | Documented evidence not meeting GS2A or GS1 criteria |
|  | **GS2A** | BP ≥ 140/90 mm Hg at 20 weeks gestation or later AND seizures leading to death. No history of epilepsy or other reason for seizures, e.g. malaria or acute encephalopthy |
|  | **GS1** | BP ≥ 140/90 mm Hg at 20 weeks gestation or later AND albuminuria ≥ 3+ AND seizures leading to death. No history of epilepsy or other reason for seizures, e.g. malaria or acute encephalopthy |
| **O64** | **OBSTRUCTED LABOUR** | |
|  | **GS4** | Unsupported clinical diagnosis |
|  | **GS3** | Documented evidence not meeting GS2A or GS1 criteria |
|  | **GS2A** | As above. Clinical diagnosis of rupture OR GS2 sepsis |
|  | **GS1** | Prolonged labour (> 12 hours) AND failure of presenting part to descend AND failure of cervix to dilate fully AND (surgical diagnosis of rupture OR GS1 sepsis) |
| **S85** | **SEPSIS** | |
|  | **GS4** | Unsupported clinical diagnosis |
|  | **GS3** | Lower abdominal tenderness and pain AND offensive discharge |
|  | **GS2A** | 1) Pyrexia ≥38oC AND 2) Lower abdominal tenderness and pain AND 3) Offensive discharge AND 4) Clinical evidence of Level 2 shock (see Appendix B) |
|  | **GS1** | As for GS2A PLUS positive blood culture |
| **BB03** | **OTHER DEFINED CAUSES OF MATERNAL DEATH** | |
| GS1-3 In assigning a level of diagnostic certainty, the same general principles apply | | |
|  | | |
|  | **INJURIES** | |
| These are external causes of injury which depend on hospital records for their verification. All are GS1 | | |
| These causes are ALWAYS considered accidental: Bite of Venomous Animal, Drowning, Falls, Fires, Poisonings, Road Traffic. They are NEVER overlapping with Homicide or Suicide. | | |
| If Homicide is indicated, it is the ONLY cause. | | |
| If Suicide is indicated, it is the ONLY cause. | | |
| **X20** | **BITE OF VENOMOUS ANIMAL** | |
| **W65** | **DROWNING** | |
| **W00** | **FALLS** | |
| **X09** | **FIRES** | |
| **T36** | **POISONINGS** | |
| **V99** | **ROAD TRAFFIC** | |
| **Y00** | **HOMICIDE** | |
| **X70** | **SUICIDE** | |
| **ZZ27** | **OTHER SPECIFIED INJURIES** | |
|  | **MISCELLANEOUS** | |
| **R99** | **UNKNOWN OR UNDEFINED** | |
|  | **FEVER** | |
|  | Axillary or oral temperature ≥38.5⁰C OR rectal temperature ≥39⁰C | |
|  | **HYPERTENSION** | |
|  | Systolic BP ≥ 140 mm Hg OR Diastolic BP ≥ 90 mm Hg | |
|  | **SHOCK** | |
|  | **GS4** | Unsupported clinical diagnosis |
|  | **GS3** | Clinical diagnosis not meeting GS1 criteria |
|  | **GS1** | Three of: |
|  |  | Obtunded |
|  |  | Heart rate > 100 |
|  |  | Respiratory rate > 20 |
|  |  | Hypotension (systolic BP <100mm Hg or a 30mm fall in baseline BP) |
|  |  | Urine output <0.5 mL/Kg/hour |
|  |  |  |

| **AIDS DEFINING CONDITIONS (CDC 2008)** |
| --- |
| Bacterial infections, multiple or recurrent* |
| Candidiasis of bronchi, trachea, or lungs |
| Candidiasis of esophagus^†^ |
| Cervical cancer, invasive^§^ |
| Coccidioidomycosis, disseminated or extrapulmonary |
| Cryptococcosis, extrapulmonary |
| Cryptosporidiosis, chronic intestinal (>1 month's duration) |
| Cytomegalovirus disease (other than liver, spleen, or nodes), onset at age >1 month |
| Cytomegalovirus retinitis (with loss of vision)^†^ |
| Encephalopathy, HIV related |
| Herpes simplex: chronic ulcers (>1 month's duration) or bronchitis, pneumonitis, or esophagitis (onset at age >1 month) |
| Histoplasmosis, disseminated or extrapulmonary |
| Isosporiasis, chronic intestinal (>1 month's duration) |
| Kaposi sarcoma^†^ |
| Lymphoid interstitial pneumonia or pulmonary lymphoid hyperplasia complex*^†^ |
| Lymphoma, Burkitt (or equivalent term) |
| Lymphoma, immunoblastic (or equivalent term) |
| Lymphoma, primary, of brain |
| *Mycobacterium avium* complex or *Mycobacterium kansasii,* disseminated or extrapulmonary^†^ |
| *Mycobacterium tuberculosis* of any site, pulmonary,^†§^ disseminated,^†^ or extrapulmonary^†^ |
| *Mycobacterium*, other species or unidentified species, disseminated^†^ or extrapulmonary^†^ |
| *Pneumocystis* *jirovecii* pneumonia^†^ |
| Pneumonia, recurrent^†§^ |
| Progressive multifocal leukoencephalopathy |
| *Salmonella* septicemia, recurrent |
| Toxoplasmosis of brain, onset at age >1 month^†^ |
| Wasting syndrome attributed to HIV |
| * Only among children aged <13 years. (CDC. 1994 Revised classification system for human immunodeficiency virus infection in children less than 13 years of age. MMWR 1994;43[No. RR-12].) |
| ^†^ Condition that might be diagnosed presumptively. |
| ^§^ Only among adults and adolescents aged >13 years. (CDC. 1993 Revised classification system for HIV infection and expanded surveillance case definition for AIDS among adolescents and adults. MMWR 1992;41[No. RR-17].) |
