# Supplementary material for: Assessing the quality of medical death certification: a case study of concordance between national statistics and results from a medical record review in a regional hospital in the Philippines
Source: Popul Health Metr. 2018 Dec 29;16:23. doi: 10.1186/s12963-018-0178-0 (PMC6311069; doi:10.1186/s12963-018-0178-0)
Supplement: Supplementary file 2 — Investigations and procedures available to patients in the Bohol Regional Hospital. Table that outlines the resources available for laboratory investigation at Bohol Regional Hospital. (DOCX 17 kb) [file 12963_2018_178_MOESM2_ESM.docx]

| **Additional file 2: Investigations and procedures available to patients in the Bohol Regional Hospital** | | | |
| --- | --- | --- | --- |
| **Type of investigation** | **List of investigations** | **Availability** | |
|  |  | **Within the hospital** | **External providers** |
| **Biochemistry** | **Liver function tests** | | |
|  | Total serum bilirubin | N | Y |
|  | Direct Bilirubin | N | Y |
|  | Indirect Bilirubin | N | Y |
|  | Urine bilirubin | Y | Y |
|  | AST (serum glutamate oxaloacetic transaminase- SGOT) | Y | Y |
|  | ALT (serum glutamic pyruvate transaminase-SGPT) | Y | Y |
|  | Serum Alkaline phosphatase | Y | Y |
|  | Prothrombin time | Y | Y |
|  | Serum albumin | Y | Y |
|  | Serum globulin | Y | Y |
|  | **Cardiac Enzymes** | | |
|  | Troponin (+/-) | N | Y |
|  | Troponin (quantitative) | N | Y |
|  | CK-MB (quantitative) | Y | Y |
|  | **Renal function tests** | | |
|  | BUN | Y | Y |
|  | Creatinine | Y | Y |
|  | **Electrolytes** | | |
|  | Serum Sodium (Na) | Y | Y |
|  | Serum Potassium (K) | Y | Y |
|  | Serum Chloride (Cl) | N | Y |
|  | Serum bicarbonate (HCO3) | N | Y |
| **Haematology** | Full blood count | Y | Y |
|  | Haematocrit | Y | Y |
|  | Haemoglobin | Y | Y |
|  | Differential count | Y | Y |
|  | Platelet count | Y | Y |
|  | I:T ratio (immature-to-total neutrophil ratio) | Y | Y |
|  | Peripheral smear | Y | Y |
| **Microbiology** | Gram staining (any specimen) | Y | Y |
|  | Bacterial cultures (any specimen) | Y | Y |
|  | AFB stain | Y | Y |
|  | Stool exam | Y | Y |
|  | Smear tests (wet mounts etc) | Y | Y |
|  | Cell count (any body fluid - CSF, pleural or peritoneal etc) | Y | Y |
|  | Malarial smear | Y | Y |
|  | HIV test – Elisa | Y | Y |
|  | HIV test – Western Blot or IFA | N | N |
|  | CD4 count | N | N |
| **Imaging** | Plain x-ray | Y | Y |
|  | CT scan | N | Y |
|  | MRI | N | N |
|  | Ultrasound | Y | Y |
|  | Echocardiography | N | Y |
| **Histopathology** | Cytopathology examination of cell blocks, smears | Y | Y |
|  | Pap smears | Y | Y |
|  | Biopsies | Y | Y |
| **Procedures** | Bone marrow biopsy | N | N |
|  | Liver biopsy | N | N |
|  | Renal biopsy | N | N |
|  | Lumbar tap | Y | Y |
|  | Blood for bacterial culture | Y | Y |
|  | General surgery/OB-Gyne procedures | Y | Y |
|  | Radiologist in hospital to read x-rays taken | Y | Y |
|  | Electrocardiogram (EKG) | Y | Y |
|  | Trained EKG reader | N | N |
|  | Spirometry | N | N |
